# Twelve Years of the Gaucher Outcomes Survey (GOS): Insights, Achievements, and Lessons Learned from a Global Patient Registry

**DOI:** 10.3390/jcm13123588

**Published:** 2024-06-19

**Authors:** Deborah Elstein, Nadia Belmatoug, Bruno Bembi, Patrick Deegan, Diego Fernandez-Sasso, Pilar Giraldo, Özlem Göker-Alpan, Derralynn Hughes, Heather Lau, Elena Lukina, Shoshana Revel-Vilk, Ida Vanessa D. Schwartz, Majdolen Istaiti, Jaco Botha, Noga Gadir, Jörn Schenk, Ari Zimran

**Affiliations:** 1 Takeda Pharmaceuticals International AG, 8152 Zurich, Switzerland; jaco.botha@takeda.com (J.B.); noga.gadir-david@takeda.com (N.G.); joern.schenk@gmx.net (J.S.); 2 Assistance-Publique Hôpitaux de Paris Nord, Université Paris Cité, 92110 Clichy, France; nadia.belmatoug@aphp.fr; 3 Centre for Lysosomal Diseases, Academic Medical Centre Hospital of Udine, 33100 Udine, Italy; brunobembi.b@gmail.com; 4 Department of Medicine, Addenbrookes Hospital, University of Cambridge, Cambridge CB2 0QQ, UK; patrick.deegan1@nhs.net; 5 Instituto William Osler, Buenos Aires C1425 BRI, Argentina; diegofernandezsasso@criocenter.com; 6 CIBER de Enfermedades Raras, IIS Aragon, 50009 Zaragoza, Spain; giraldocastellano@gmail.com; 7 Translational Research Unit, IIS Aragon, 50009 Zaragoza, Spain; 8 Lysosomal Disorders Unit and Center for Clinical Trials, O and O Alpan LLC, Fairfax, VA 22030, USA; ogoker-alpan@ldrtc.org; 9 Lysosomal Storage Disorders Unit, Department of Haematology, Royal Free Hospital, UCL Medical School, London NW3 2QG, UK; derralynnhughes@nhs.net; 10 Langone Medical Cessnter, New York University, New York, NY 10016, USA; 11 Department of Orphan Diseases, National Medical Research Center for Hematology, 125167 Moscow, Russia; elenalukina02@gmail.com; 12 Gaucher Unit, The Eisenberg R&D Authority, Shaare Zedek Medical Center, Jerusalem 9103102, Israel; srevelvilk@gmail.com (S.R.-V.); joleenist@szmc.org.il (M.I.); azimran@gmail.com (A.Z.); 13 School of Medicine, Hebrew University, Jerusalem 9112102, Israel; 14 Genetics Department, Federal University of Rio Grande do Sul (UFRGS), Medical Genetics Service—Clinic Hospital of Porto Alegre, Porto Alegre 90010-150, Brazil; idadschwartz@gmail.com

**Keywords:** velaglucerase alfa, enzyme replacement therapy, ERT, Gaucher disease, GOS, registry

## Abstract

**Background:** Long-term patient registries are important for evaluating treatment outcomes in patients with rare diseases, and can provide insights into natural disease history and progression in real-world clinical practice. Initiated in 2010, the Gaucher Outcome Survey (GOS) is an ongoing, international, multicenter, observational registry (ClinicalTrials.gov Identifier: NCT03291223) for patients with a diagnosis of Gaucher disease (GD), irrespective of treatment type or status, with a primary objective to monitor safety and long-term effectiveness of velaglucerase alfa. **Methods:** Here, we evaluated the GOS population 12 years after the registry initiation. **Results:** As of 25 February 2023, 2084 patients enrolled in the GOS and 1643 received GD-specific treatment. Patients exhibited broad heterogeneity at baseline: age of diagnosis (0 to 85.3 years), hemoglobin concentrations (<80.0 g/L to >150 g/L), platelet counts (<50 × 10^9^/L to >450 × 10^9^/L), and liver and spleen volumes. Most patients treated with enzyme replacement therapy or substrate reduction therapy reported improvements in clinical parameters within 1 year of treatment initiation, maintained over the course of treatment up to 12 years, whereas untreated patients had baseline values closer to standard reference thresholds and showed stability over time. **Conclusion:** The 12-year data from the GOS confirm the impact of long-term treatment with GD-specific agents and offer insights into disease progression and outcomes in a real-world setting.

## 1. Introduction

Gaucher disease (GD) results from an autosomal recessive inherited deficiency of the lysosomal enzyme β-glucocerebrosidase caused by pathogenic mutations in the *GBA1* gene, which lead to accumulation of storage materials, predominantly the sphingolipid glucocerebroside, in cells of the monocyte–macrophage system [1]. Typical clinical manifestations of GD include splenomegaly, hepatomegaly, bone pain, thrombocytopenia, anemia, and various skeletal disorders [1]. GD manifests with considerable heterogeneity, ranging from asymptomatic to severe disease; although the wide spectrum of disease manifestations can be regarded as a phenotypic continuum [2], patients are typically categorized into three disease types (1, 2, and 3) based on presence (GD2 and GD3) or absence (GD1) of early neurological manifestations in childhood. Clinical trials have demonstrated that enzyme replacement therapy (ERT) and substrate reduction therapy (SRT) can result in improvements in the systemic manifestations of GD [3,4,5,6]; however, neither ERT nor SRT have been proven to have an effect on GD-associated neurological manifestations [1]. Furthermore, insufficient understanding of the natural history of the disease as a result of the phenotypic and geographic diversity of the small disease population may affect the development of effective management strategies and hamper the selection of meaningful assessments relevant to patients and physicians [7].

Long-term patient registries are effective means of assessing treatment outcomes, impacts on disease manifestations, and real-world utilization of therapies in patients with rare diseases. In contrast to clinical trials, which by definition are limited to small patient numbers in rare diseases, rare disease registries afford the opportunity to amass real-world longitudinal data from larger cohorts, including children, pregnant women, asymptomatic patients, and patients with neuronopathic GD, over longer time periods, while collection of cross-sectional registry data allow the reconstruction of natural histories, which would otherwise not be evident in rare disorders [8]. A number of international registries for patients with GD exist, the largest being the International Collaborative Gaucher Group disease registry (ClinicalTrials.gov Identifier: NCT00358943), initiated by Genzyme in 1991 (now Sanofi), followed by the Gaucher Outcome Survey (GOS) disease registry, initiated in 2010 by Shire (now Takeda) after US Food and Drug Administration approval of velaglucerase alfa, and a drug-specific registry Taliglucerase Alfa Surveillance, for patients receiving treatment with taliglucerase alfa, initiated by Pfizer in 2013 (EUPAS4721 [9]), as well as national registries including the Spanish Gaucher Disease Registry, initiated in 1993 [10], the French Gaucher Disease Registry, initiated in 2009 [11], and the GAUCHERITE registry initiated in 2014 in the United Kingdom [12]. 

The GOS is an ongoing, international, observational, long-term, disease-specific registry for patients with a confirmed GD diagnosis, regardless of their GD type or treatment status [13,14,15,16,17]. The GOS provides an opportunity to evaluate patient characteristics, treatment patterns, clinical outcomes, and medical events in patients with GD in a “real-world” setting. Here, we review 12 years of data from the GOS and discuss the contributions that data from the GOS have made to the overall knowledge of GD.

## 2. Materials and Methods

### Registry Design and Objectives

The GOS is a long-term global registry, registered as a phase 4 post-marketing observational study (ClinicalTrials.gov Identifier: NCT03291223), designed to collect information on patients with confirmed GD from data obtained during routine patient visits and assessments. The registry is open to patients of any age, sex, or ethnic origin with a confirmed diagnosis (biochemical and/or genetic) of GD, who are not participating in ongoing blinded clinical trials. Written informed consent was obtained from each patient. For patients <18 years of age (<16 years of age in the United Kingdom), consent was obtained from a parent or legal representative, along with assent where appropriate. All patient data were collected and handled in accordance with the study protocol, relevant global and local regulations, best practice, and with Good Pharmaco-epidemiological Practice, Good Research for Comparative Effectiveness principles, and the principles of the International Conference on Harmonization Good Clinical Practice guidelines. The registry is operated by Shire (now Takeda) with a validation process monitored by a contracted research organization (PPD Global Ltd., Cambridge, UK). The monitoring focused on safety events, informed consent forms, inclusion/exclusion criteria, and verification of all data points in a random group of patients (approximately 30% of total patients). The primary objectives of the GOS are to monitor the safety and long-term effectiveness of velaglucerase alfa. Secondary objectives are to characterize treated (receiving velaglucerase alfa or other GD-specific treatments) and untreated patients with GD, to gain a better understanding of the natural history of GD and to serve as a database for evidence-based management of GD over time in real-life clinical practice. 

Data are collected on a broad range of parameters, including patient demographics, physical characteristics, medical history, hematologic and visceral parameters, biomarkers, bone imaging, adverse events (AEs), and GD-specific treatment status via web-based electronic case report forms (eCRFs) by authorized personnel at each site. A medical history module unique to the GOS (as discrete categories by organ system) was designed to inform about associated diseases and comorbidities. AEs occurring during the study period are recorded and coded using the Medical Dictionary for Regulatory Activities coding dictionary. Serious AEs are defined as any event or reaction that results in death, a life-threatening AE, inpatient hospitalization, or prolongation of existing hospitalization. Several mandatory fields are included in the GOS eCRF to ensure that records from each patient are analyzable for basic parameters. The quality of data entered into the GOS is automatically checked using predefined, established limits set within the registry database program. Additional data checks are performed by the data managers and site monitors to detect inconsistencies. If abnormal, confounding, or missing data (due to a parameter not being measured or recorded) are detected for a database entry, a request for clarification is electronically sent to the investigator’s site. 

The eCRF modules for both spleen and liver allow for organ volume data provided by ultrasound, magnetic resonance imaging (MRI), or computed tomography (CT). Multiple of normal (MN; normalized to body weight) was also determined: liver MN of 1.0 was calculated as 2.5% of body weight and spleen MN of 1.0 was calculated as 0.2% of body weight. Imaging modalities included plain radiographs (X-rays), ultrasound, MRI, and CT. Bone mineral density was assessed by dual-energy X-ray absorptiometry at the lumbar spine and the femoral neck, and reported as T-scores. Medical history events were captured in the eCRF as free-text and subsequently categorized into 22 categories based on organ systems or areas of pathology (Table S1). 

Descriptive statistics were calculated for demographic and outcome data. Baseline was defined as the start of treatment (including treatment started prior to enrollment in the GOS), or date of enrollment into the GOS for untreated patients, to allow evaluation of change from baseline. Untreated patients were defined as patients who were untreated at enrollment and remained untreated at the last follow-up. Medical history data were analyzed by age category (<18 years vs. ≥18 years), sex, geographic location, genetic mutation, and splenectomy status. Patients who underwent a partial splenectomy were included in the non-splenectomized group, as owing to re-growth of the splenic remnant, patients who underwent partial splenectomy were comparable to those with intact spleens with regard to the hematologic and visceral disease trajectory and the impact of disease-specific therapies [18,19]. All statistical analyses were performed using SAS 9.4 (SAS Institute Inc., Cary, NC, USA). No between-group statistical comparisons were performed.

## 3. Results

### 3.1. Patient Characteristics

As of 25 February 2023, there were 2084 patients enrolled in the GOS, from 47 centers in 17 countries. Of these, 1135 (54.5%) were female, 1404 (67.4%) were ≥18 years old at enrollment, and 259 (12.4%) had undergone a total splenectomy either prior to or during enrollment in the GOS (Table 1); 71 (3.4%) patients had undergone partial splenectomy. The Ashkenazi Jewish population was the most frequently reported ethnic group in the GOS registry (53.6%). Based on clinical assessment with biochemical and/or genetic confirmation of GD, most patients (1862 [93.6%]) were diagnosed with GD1, 10 (0.5%) with GD2, and 118 (5.9%) with GD3 (GD type was unavailable for 94 patients). Genotype information was available for 1481 (71.1%) patients, the majority of whom had at least one c.1226A>G (p.Asp409Ser) allele (Table 1). All six patients with c.1342G>C (p. Asp448His) homozygous genotype had a recorded clinical diagnosis of GD3. Of the 134 patients with a known c.1448T>C (p.Leu483Pro) homozygous (*n* = 76) or heterozygous with other mutation (*n* = 58) genotype, 79 (59.0%) had a recorded clinical diagnosis of GD3, 2 (1.5%) of GD2, 46 (34.3%) of GD1, and 7 (5.2%) were not classified. A list of mutations identified as ‘other’ is provided in Table S1.

### 3.2. Treatment Patterns

**Figure 1:**
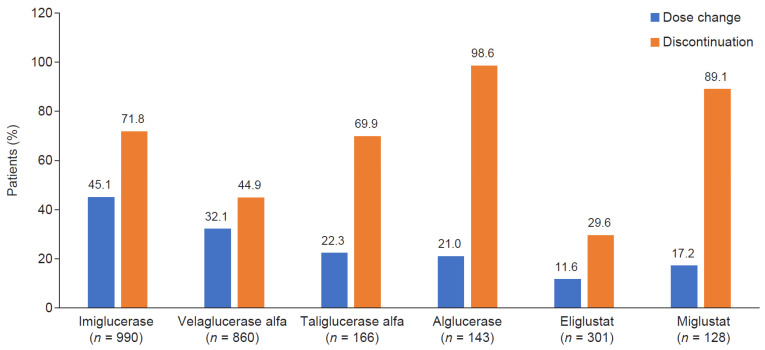
Proportion of patients with dose change or discontinued treatment.

Data captured in the GOS can provide insights into treatment patterns with GD-specific treatments. As of 25 February 2023, a total of 1643 patients (78.8%) enrolled in the GOS had received at least one dose of any GD-specific treatment, and 441 patients enrolled and received no treatment. The most frequently used treatments were imiglucerase (990 [47.5%] patients) and velaglucerase alfa (886 [42.5%] patients). For 886 patients receiving velaglucerase alfa at any time while enrolled in the GOS, this agent was most widely used at 45–60 U/kg (40.8% of patients with available data) and every other week (94.4%) (Table 2), but there were differences in dosing between the three highest-enrolling countries, with median doses of 30 U/kg in Israel, 41 U/kg in the United Kingdom, and 60 U/kg in North America. The greatest proportions of dose changes were recorded for 446 (45.1%) patients receiving imiglucerase and 276 (32.1%) patients receiving velaglucerase alfa, and the greatest proportion of treatment withdrawals occurred with alglucerase (141 of 143 [98.6%] patients with available data), which was discontinued from use after approval of imiglucerase in 1994, followed by miglustat (114 of 128 [89.1%] patients) (Figure 1). 

### 3.3. Past Medical History

**Figure 2:**
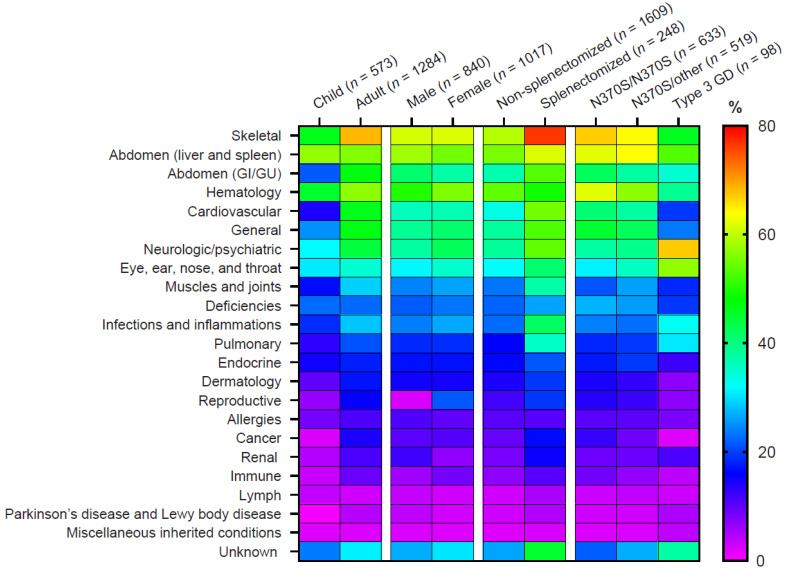
Medical history. Abdomen category includes liver and spleen volume; hematology category includes hemoglobin concentrations and platelet counts (Table S2). GI, gastrointestinal; GU, genitourinary.

A total of 18,772 medical conditions were recorded in the medical history records of 1857 patients enrolled in the GOS. Medical history events were reported at similar rates for males and females across most categories, with the exception of reproductive disorders, but more conditions were reported for adults than children, consistent with an increasing prevalence of morbidities with age in the general population [20], and for treated patients than untreated patients. The most prevalent medical history events overall were consistent with the predominant disease manifestations of GD: skeletal involvement was recorded for 1143 (61.6%) patients, abdominal involvement (including hepatomegaly and splenomegaly) for 1048 (56.4%) patients, and hematologic involvement (including anemia and thrombocytopenia) for 988 (53.2%) patients. The most prevalent medical history events for patients with a clinical diagnosis of GD3 were neurological/psychiatric events (66 [67.3%] patients), consistent with the presence of neurological features, with a high frequency of eye, ear, nose, and throat (56 [57.1%] patients); abdominal (52 [53.1%] patients); and skeletal (45 [45.9%] patients) events. Patients who had undergone a total splenectomy reported more medical history events than non-splenectomized patients, most notably cardiovascular events (reported by 55.2% splenectomized vs. 33.6% non-splenectomized patients), infections and inflammation (41.9% vs. 22.7% patients), pulmonary events (35.5% vs. 16.0% patients), and skeletal events (76.6% vs. 59.2% patients). Cancer was reported for 196 (10.6%) patients overall: 183 (14.3%) adults and 13 (2.3%) children. More patients reporting cancer or tumors had the c.1226A>G (p.Asp409Ser)/c.1226A>G (p.Asp409Ser) genotype compared with c.1226A>G (p.Asp409Ser)/other mutation and c.1448T>C (p.Leu483Pro)-containing genotypes (12.5% vs. 9.1% and 3.6% of patients (Figure 2). 

### 3.4. Clinical Response to Treatment

**Figure 3:**
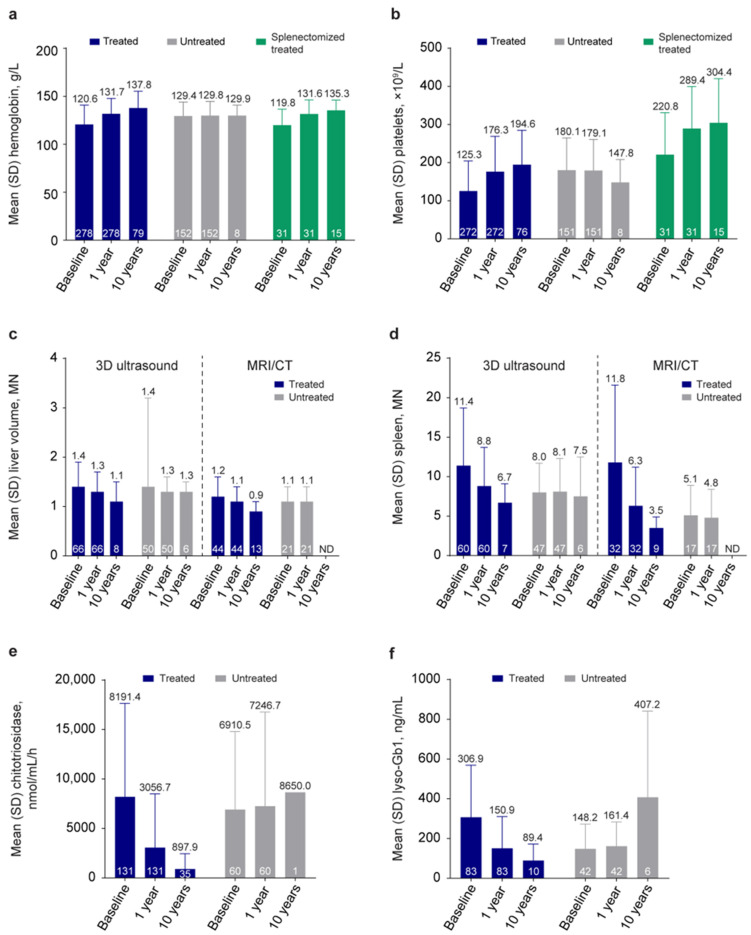
Clinical parameters and biomarkers over time. (**a**) Hemoglobin concentrations and (**b**) platelet counts at baseline, 1 year, and 10 years for treated, untreated, and splenectomized-treated patients. (**c**) Liver volume and (**d**) spleen volume at baseline, 1 year, and 10 years for treated and untreated patients assessed using 3D ultrasound and MRI/CT. (**e**) Chitotriosidase and (**f**) lyso-Gb1 biomarkers at baseline, 1 year, and 10 years for treated and untreated patients. Baseline is defined as the date of treatment initiation for treated patients and the date of GOS entry for untreated patients. The numbers inside the bars indicate the number of patients analyzed. The numbers above the bars are the means.

The impact of treatment on disease progression can be assessed using data entered in the GOS for routinely captured clinical outcomes. Of 2084 patients in the GOS at 12 years, 278 and 272 treated patients and 152 and 151 untreated patients reported hemoglobin concentrations and platelet counts, respectively, at both baseline and at least one post-baseline time point. Mean (SD) hemoglobin concentrations improved from 120.6 (20.2) g/L at baseline to 131.7 (16.1) g/L after 1 year of treatment (*n* = 278), and were maintained long-term (Figure 3). The proportion of treated patients with hemoglobin concentrations at or above the standard reference threshold, defined as ≥110.0 g/L (children ≤12 years of age and females) or ≥120.0 g/L (males) [21,22], increased from 70.1% at baseline to 92.8% after 1 year, and remained consistently above 90% for up to 12 years. Patients with c.1448T>C (p.Leu483Pro)-containing genotypes had lower hemoglobin concentrations at baseline than c.1226A>G (p.Asp409Ser)-containing genotypes, but neither genotype nor splenectomy status affected the magnitude of change in hemoglobin concentration over time (Figure 3).

Similarly, mean (SD) platelet counts improved from 125.3 (79.1) × 10^9^/L at baseline to 176.3 (92.6) × 10^9^/L after 1 year of treatment (*n* = 272), and were maintained long-term (Figure 3); 40.8% of patients had platelet counts at or above the acceptable goal of GD treatment (≥120 × 10^9^/L [21,22]) at baseline, increasing to 68.7% at 1 year, and remaining above 80% from year 2 throughout the follow-up period. Splenectomy status appeared to affect platelet counts at baseline but had no impact on response to treatment. Treated patients who had undergone total splenectomy had higher baseline platelet counts than non-splenectomized patients (mean, 220.8 × 10^9^/L [*n* = 31] vs. 113.0 × 10^9^/L [*n* = 241], respectively), but improvements in both cohorts were observed after 1 year of treatment (mean, 289.4 × 10^9^/L vs. 161.8 × 10^9^/L at 1 year). Baseline platelet concentrations were similar irrespective of genotype, and the percentage of patients achieving the GD treatment goal of ≥120 × 10^9^/L increased from baseline with each genotype, and was achieved by most patients after 1 year of treatment (34.8% to 60.0% with c.1226A>G (p.Asp409Ser)/c.1226A>G (p.Asp409Ser), 38.1% to 66.7% with c.1226A>G (p.Asp409Ser)/other mutation, and 32.0% to 72.0% with c.1448T>C (p.Leu483Pro)/other mutation). Patients with c.1448T>C (p.Leu483Pro)-containing genotypes saw the greatest improvement from baseline to 1 year (from 119.4 × 10^9^/L to 209.2 × 10^9^/L).

Long-term improvements in liver and spleen volumes were observed with treatment. After 1 year, the mean (SD) liver volume reduced from 1.4 (0.5) to 1.3 (0.4) MN when assessed by 3D ultrasound (*n* = 66), and from 1.2 (0.4) to 1.1 (0.3) MN when assessed with CT/MRI (*n* = 44), all maintained long-term. Similarly, mean (SD) spleen volume reduced from 11.4 (7.3) to 8.8 (4.9) MN using 3D ultrasound (*n* = 60), and 11.8 (9.8) to 6.3 (4.9) MN using CT/MRI (*n* = 32), both maintained long-term (Figure 3). 

In contrast to treated patients, most patients remaining untreated had hematologic and visceral parameters close to or above GD treatment targets at baseline that remained stable throughout GOS enrollment, indicating mild disease severity not requiring pharmacological intervention. Untreated patients with available data had a mean (SD) baseline hemoglobin concentration of 129.4 (14.6) g/L (*n* = 152) and a mean (SD) baseline platelet count of 180.1 (84.6) × 10^9^/L (*n* = 151), both of which remained stable over time (Figure 3). Similarly, mean baseline liver and spleen volumes for untreated patients were similar to those for treated patients after 1 year of treatment (Figure 3). 

### 3.5. Biomarker Analysis

Of the range of available biomarkers for GD, few were measured routinely or repeatedly in real-world clinical practice over the duration of the study. However, data for chitotriosidase activity and glucosylsphingosine (lyso-Gb1) concentration were available for 131 (7.8%) and 83 (5.1%) treated patients and 60 (13.6%) and 42 (9.5%) untreated patients at baseline, respectively. The number of patients with post-baseline biomarker assessments reduced over time, primarily owing to variations in the frequency of follow-up visits and repeat assays. Nonetheless, chitotriosidase activity was shown to reduce rapidly in patients receiving treatment, with a 35.4% reduction achieved at 1 year (*n* = 128) and >75% at 5 years, maintained up to 20 years (Figure 3). Similar reductions were observed with lyso-Gb1, with a 47.2% reduction achieved at year 1 (*n* = 83) that was maintained long-term (Figure 3). 

### 3.6. Skeletal Abnormalities

Of 2084 patients in the GOS at 12 years, 805 (49.0%) treated patients and 66 (15.0%) untreated patients reported any bone imaging results, with an average of 5.8 and 10.4 scans (CT, MRI, ultrasound, X-ray) per patient, respectively. A total of 3511 and 578 MRI scans, 1084 and 98 X-rays, 53 and 2 CT scans, and 2 and 0 ultrasound scans each were carried out in treated and untreated patients, respectively, for the purpose of orthopedic imaging. The imaging method was not recorded for 48 scans. More scans were carried out in splenectomized versus non-splenectomized patients (7.6 vs. 5.8 scans per patient, respectively). Imaging revealed a total of 5376 bone abnormalities in 871 patients, of which 1639 (30.5%) were bone marrow infiltration, 711 (13.2%) were Erlenmeyer flask deformities, 371 (6.9%) were avascular necrosis, and 95 (1.8%) were fractures (see Table 3 for differences between treated and untreated patients). Overall, 329 (47.0%) and 283 (44.9%) adults aged ≥21 years showed normal lumbar spine and femoral neck bone density, respectively, using dual-energy X-ray absorptiometry and based on T-score measurements (T-score ≥ −1.0). Osteoporosis (T-score ≤ −2.5) was detected in the lumbar spine and femoral neck for 97 (13.9%) and 57 (9.0%) adults aged ≥21 years, respectively (Table 3). 

### 3.7. Pregnancy Outcomes

Of 1135 female patients in the GOS at 12 years (896 treated, 239 untreated), 400 patients reported a total of 932 pregnancies at any time. Overall, most pregnancies resulted in live births, with no reported impact of GD-specific treatment (671 [86.5%] vs. 138 [88.5%] for treated vs. untreated patients, respectively). Overall, 51 (12.8%) patients reported 62 events of spontaneous pregnancy loss: 44 (13.2%) treated and 7 (10.4%) untreated patients.

### 3.8. AEs Summary

Of 2084 patients enrolled in the GOS, 581 (27.9%) reported a total of 1499 AEs, and 364 (17.5%) reported a total of 815 serious AEs (SAEs). Of the AEs, 41 events in 29 patients were considered related to velaglucerase alfa, including 15 events of infusion-related reaction in 11 patients (Table 4). AEs were more frequent in treated versus untreated patients (33.6% vs. 6.6%, respectively). The most frequently reported AEs (regardless of relationship to treatment) were COVID-19 (42 [2.0%]), arthralgia (29 [1.4%]), headache (24 [1.2%]), back pain (23 [1.1%]), and abdominal pain (22 [1.1%]). There were 104 recorded fatal AEs and 89 deaths overall (4.3% of patients): 48 (53.9%) males and 41 (46.1%) females. Of these, 76.4% of deaths occurred in patients aged ≥60 years (range, 0.2–93.1 years at time of death). The main causes of death were cancer (19 [21.3%]), respiratory disease (12 [13.5%]), and cardiac disorders (7 [7.9%]). A total of 15 deaths (16.9%) were reported with causes unknown or missing. In total, 11 deaths were children, including 6 children aged <5 years diagnosed with GD2, 4 aged ≤5 years with no GD type recorded, and 1 aged <12 years who had a diagnosis of GD3. Causes of death in children were respiratory failure or pneumonia (*n* = 6; 4 GD2 and 2 no GD type reported), GD progression including neuronopathic symptoms (*n* = 4; 2 GD2, 1 GD3, 1 no GD type reported), and multiple organ dysfunction syndrome (*n* = 1; no GD type reported). 

## 4. Discussion

This analysis of the GOS population at 12 years since initiation of the GOS registry showed broad heterogeneity in patient demographics and clinical characteristics at baseline, encompassing older patients >65 years of age with mild symptoms who remained untreated, through adolescents and young adults with more severe symptoms of GD1, to young pediatric patients with severe neurological forms of GD. Most (79%) of the patients were treated with ERT or SRT and reported improvements in clinical parameters within 1 year of treatment initiation, which was maintained over the course of treatment up to 12 years. Untreated patients had baseline values closer to standard reference thresholds and showed stability over time. Medical history events occurred more frequently in treated versus untreated patients. Patients who underwent total splenectomy had more skeletal, infections, and pulmonary medical history events versus non-splenectomized patients.

In this analysis, increases in hemoglobin concentrations and platelet counts, and reductions in liver and spleen volume and biomarker concentrations over time were observed in patients receiving GD-specific treatments for up to approximately 12 years, resulting in a large proportion of patients achieving and maintaining values at or above standard reference or diagnostic thresholds for anemia, thrombocytopenia, and hepatosplenomegaly. Smaller changes over time were observed in untreated patients compared with treated patients, although baseline values were typically closer to accepted reference clinical thresholds for untreated patients. Consistent with previous studies [23,24,25], platelet counts were markedly higher at all time points for splenectomized patients versus non-splenectomized patients, both treated and untreated, while splenectomy status had no clear effect on hemoglobin concentrations in patients receiving treatment. 

A strength of the GOS is the inclusion of comprehensive medical history data for patients with GD. Overall, medical history events occurred at higher rates in patients who subsequently received GD-specific treatment compared with patients who remained untreated, likely to be reflective of greater disease severity in treated patients. Furthermore, patients with more severe disease could be expected to have more frequent medical visits, improving the likelihood of detection of coexisting conditions. Fewer medical history events were reported in children compared with adults, possibly related to fewer medical visits, insufficient time for development or detection of events, and the absence of age-related complaints, and warrants further investigation in relation to a healthy population.

As expected, the most prevalent medical history categories reported in this analysis were consistent with the predominant manifestations of the disease: abdominal (liver and spleen volume), skeletal and hematologic categories, and neurologic symptoms for patients with GD3. The frequency of skeletal, infections, and pulmonary medical history events was greater for patients who had undergone total splenectomy (used in the past to ameliorate severe hypersplenism) versus non-splenectomized patients (including patients who had a partial splenectomy, owing to potential for re-growth of the splenic remnant), consistent with previously published data indicating that splenectomy is associated with an acceleration of the progression of skeletal disease and risk of morbidities [26,27,28,29,30,31]. Bone imaging data revealed that the occurrence of skeletal abnormalities after diagnosis were more frequent in splenectomized patients compared with non-splenectomized patients.

In agreement with previous GOS data [32], the proportion of pregnancies with normal outcomes were similar with or without treatment for GD, and rates of spontaneous abortion were similar to reported rates in the healthy general population (11–22%) [33,34]. High levels of medical surveillance may contribute to these findings, although reporting bias, with incomplete reporting of spontaneous abortions, cannot be discounted. 

AEs reported in this analysis were consistent with those from clinical trials of ERT, with no new safety signals identified from registry data [5,14,15,35,36,37]. The overall number of events was higher in treated versus untreated patients, possibly reflective of more frequent healthcare visits or greater disease severity warranting treatment; however, the proportion of events considered to be related to velaglucerase alfa treatment was low, with SAEs related to velaglucerase alfa occurring in two (0.2%) patients. The higher rate of AEs observed in the United Kingdom compared with other regions warrants suspicion of reporting bias or different interpretation of AEs, and justifies careful monitoring. 

Since the initiation of the GOS, the registry has evolved to keep pace with developments in the management of patients with GD, to reflect current practice, updated assessment methodologies, and development of new biomarkers. However, to the greatest extent, the success of a registry is dependent on the quantity and quality of information inputted by participating physicians, and the willingness of individuals to provide information for entry into the database. This includes the lack of information on GD type in 94 patients, which was not recorded in the GOS registry. An early limitation of the GOS registry was the initial misapprehension that only treated patients, especially those treated with velaglucerase alfa, should be enrolled. Nonetheless, the number of patients treated with imiglucerase enrolled in the GOS has increased over time and is now comparable to the number of patients receiving velaglucerase alfa. The greatest proportion of patients enrolled in GOS were from Israel and the United States, perhaps owing to recruitment of patients from these countries into velaglucerase alfa clinical trials and subsequent enrollment into the GOS. This has resulted in an overrepresentation of individuals with Ashkenazi Jewish heritage, who have a high frequency of the comparatively mild c.1226A>G (p.Asp409Ser) genotype, while patients from Asian and Middle Eastern countries, where typically there are high frequencies of GD2 and GD3, are underrepresented in the GOS. Further regional differences include a relatively high proportion of untreated or low dose–treated patients in Israel compared with other regions, which may be reflective of milder disease severity associated with the predominant c.1226A>G (p.Asp409Ser)/c.1226A>G (p.Asp409Ser) genotype, as well as prescribing practices based on studies indicating good outcomes with low doses [38,39,40,41], local guidelines, regulations, and reimbursement factors. 

As with any registry, data are collected during routine clinical practice; therefore, the frequency of visits, and the assessments carried out at each visit, can vary considerably among patients. As a result, data records may be incomplete or misaligned, with missing data impacting on the strength of analyses possible. Within this analysis, several examples of likely or confirmed data-entry errors are apparent, most notably for one patient included in the untreated cohort, who was later determined to have received treatment, but for whom treatment-related data were missing from the database. Other possible data-entry errors included incorrect unit selection and misplacement of a decimal place. Although it is not possible to account for all data-entry errors, highly improbable outliers were excluded from analyses, and sensitivity analyses found that the impact of possible or probable data-input errors was negligible to the overall result. In addition, the GOS registry had a validation process in place that mostly monitored the safety events but also verified other data points. 

Important limitations pertinent to data evaluation raise concerns regarding non-standardization of clinical and biochemical assessments among centers or countries. This is notable for measurement of GD-specific biomarkers, which show considerable variation among laboratories, assessment of liver and spleen volumes, and skeletal abnormalities. The methodologies employed, each with their own strengths and weaknesses, could potentially impact the type and extent of abnormalities observed in different patients, and although analyses can be separated by centers or by methodology, this has the effect of further reducing sample sizes. Evaluation of the impact of therapy on GD-specific biomarkers was limited in this study due to the small number of patients with biomarker concentrations at baseline and the variability in the collection of post-baseline data. Nonetheless, our data are consistent with previously reported decreases in lyso-Gb1 levels following treatment with velaglucerase alfa [42]. Additionally, the use of free-text fields for the input of medical histories and the lack of clear separation from disease manifestations introduces additional challenges in the categorization of medical history events. Assessments must also take into consideration that dosing of GD-specific treatments may differ among centers and regions, including regional differences in recommended dosing regimens or local guidance, and also among patients, perhaps owing to disease severity or perceived tolerance of treatments. Furthermore, the definition of baseline as the date of GOS enrollment for untreated patients fails to account for differences in time since symptom onset and disease severity and may introduce further variability.

## 5. Conclusions

Over 12 years, the GOS has contributed to information on the characterization of patients with GD, including GD3, to help guide the evidence-based management of patients with the disease. The registry has enabled in-depth evaluation of natural history, disease progression, geographic variances in treatment dosing, clinical outcomes, safety outcomes, patient subgroups, relevance of biomarkers, and more, which would otherwise be challenging to investigate in a clinical trial setting. The current report provides useful data relating to long-term treatment of patients with different GD-specific agents, and potential insights from medical history data into disease progression and outcomes in a real-world setting. These findings underscore the importance of real-life experience in countries with varying patient population demographics, multiple treatment options, and importantly, with complex medical comorbidities that may or may not be directly associated with deficiency of the enzyme β-glucocerebrosidase. 

## Figures and Tables

**Table 1:** Patient demographics at the time of entry into the GOS.

	Treated (*n* = 1643)	Untreated (*n* = 441)	Total (*N* = 2084)
Sex			
*n*	1643	439	2082
Male, *n* (%)	747 (45.5)	200 (45.6)	947 (45.5)
Female, *n* (%)	896 (54.5)	239 (54.4)	1135 (54.5)
Age category			
*n*	1643	439	2082
<18 years, *n* (%)	548 (33.4)	130 (29.6)	678 (32.6)
≥18 years, *n* (%)	1095 (66.6)	309 (70.4)	1404 (67.4)
Age at diagnosis, years *			
*n*	1500	340	1840
Mean (SD)	19.1 (17.3)	23.9 (18.9)	20.0 (17.7)
Median (range)	14.6 (0–85.3)	21.5 (0–78.9)	16.0 (0–85.3)
Age at baseline, years			
*n*	1643	439	2082
Mean (SD)	30.1 (20.1)	34.2 (21.9)	31.0 (20.6)
Median (range)	29.2 (0.1–87.2)	32.9 (0.1–81.6)	30.0 (0.1–87.2)
Age at GOS entry, years *			
*n*	1643	439	2082
Mean (SD)	39.0 (20.7)	34.2 (21.9)	38.0 (21.0)
Median (range)	38.1 (0–90.9)	32.9 (0.1–81.6)	37.0 (0–90.9)
Splenectomized (total), *n* (%)	237 (14.4)	22 (5.0)	259 (12.4)
Ethnicity			
*n*	1345	321	1666
Ashkenazi Jewish, *n* (%)	634 (47.1)	259 (80.7)	893 (53.6)
Other, *n* (%)	711 (52.9)	62 (19.3)	773 (46.4)
Geographic region, *n* (%)			
North America ^†^	549 (33.4)	107 (24.3)	656 (31.5)
Israel	546 (33.2)	290 (65.8)	836 (40.1)
United Kingdom	148 (9.0)	11 (2.5)	159 (7.6)
Rest of world	400 (24.3)	33 (7.5)	433 (20.8)
GD type			
*n*	1589	401	1990
1, *n* (%)	1473 (92.7)	389 (97.0)	1862 (93.6)
2, *n* (%)	4 (0.3)	6 (1.5)	10 (0.5)
3, *n* (%)	112 (7.0)	6 (1.5)	118 (5.9)
*GBA1* genotype			
*n*	1126	355	1481
c.1226A>G (p.Asp409Ser)/c.1226A>G (p.Asp409Ser), *n* (%)	428 (38.0)	258 (72.7)	686 (46.3)
c.1226A>G (p.Asp409Ser)/other mutation ^‡^, *n* (%)	497 (44.1)	74 (20.8)	571 (38.6)
c.1448T>C (p.Leu483Pro)/c.1448T>C (p.Leu483Pro) ^§^, *n* (%)	71 (6.3)	5 (1.4)	76 (5.1)
c.1448T>C (p.Leu483Pro)/other mutation ^¶^, *n* (%)	55 (4.9)	3 (0.8)	58 (3.9)
c.1342G>C (p.Asp448His)/c.1342G>C (p.Asp448His), *n* (%)	5 (0.4)	1 (0.3)	6 (0.4)
Other mutation ^∥^, *n* (%)	70 (6.2)	14 (3.9)	84 (5.7)
Duration in GOS, years			
Mean (SD)	6.1 (3.1)	5.1 (3.2)	5.9 (3.2)
Median (range)	6.1 (0–12.2)	5.0 (0–11.8)	5.7 (0–12.2)

Abbreviations: GD, Gaucher disease; GOS, Gaucher Outcome Survey. Baseline is defined as the start of treatment for treated patients, or enrollment in the GOS for untreated patients. * Includes patients diagnosed and enrolled in the GOS at birth, owing to family history of GD. ^†^ Includes Canada and the United States. ^‡^ Includes c.1226A>G (p.Asp409Ser)/c.1448T>C (p.Leu483Pro). ^§^ Patients identified as GD2 or GD3. ^¶^ Excludes c.1448T>C (p.Leu483Pro)/c.1226A>G (p.Asp409Ser). ^∥^ Table S1 for more details on these mutations.

**Table 2:** Disease-specific treatment dosages and frequencies (patients who received more than one dose).

	Starting Dose	Most Common Dose Category (At Starting Dose)		Most Common Dose Frequency (At Starting Dose)	
ERT	Median (Range), U/kg	Dose, U/kg	Patients, *n* (%)	Dose	Patients, *n* (%)
Imiglucerase (*n* = 958)	30.0 (2.0–120.0)	15–30	317 (33.1)	EOW	865 (90.3)
Velaglucerase alfa (*n* = 843)	43.0 (5.20–195.1)	45–60	344 (40.8)	EOW	795 (94.4) *
Taliglucerase alfa (*n* = 165)	30.0 (15.0–120.0)	15–30	80 (48.5)	EOW	163 (98.8)
Alglucerase (*n* = 134)	30.0 (1.0–150.0)	15–30	62 (46.3)	EOW	102 (77.3) ^†^
**SRT**	**Median (Range), mg**	**Dose, mg**	**Patients, *n* (%)**	**Dose**	**Patients, *n* (%)**
Eliglustat (*n* = 290)	84 (30–300)	84	268 (92.4)	BID	239 (82.7) ^‡^
Miglustat (*n* = 123)	100 (50–300)	100–300	109 (89.3) ^§^	TID	101 (82.1)

Abbreviations: BID, twice daily; EOW, every other week; ERT, enzyme-replacement therapy; SRT, substrate reduction therapy; TID, three times a day. * Dose frequency data were missing in one patient. ^†^ Dose frequency data were missing in two patients. ^‡^ Dose frequency data were missing in one patient. ^§^ Dose category data were missing in one patient.

**Table 3:** Summary of skeletal abnormalities.

	Treated (*n* = 1643)	Untreated (*n* = 441)	Total (*N* = 2084)
Orthopedic imaging categories, *n* (%)			
Bone marrow infiltration	1371 (29.2)	268 (39.1)	1639 (30.5)
Erlenmeyer flask deformity	623 (13.3)	88 (12.8)	711 (13.2)
Avascular necrosis	354 (7.5)	17 (2.5)	371 (6.9)
Lytic lesions	107 (2.3)	4 (0.6)	111 (2.1)
Fractures	92 (2.0)	3 (0.4)	95 (1.8)
Osteoarthritis	76 (1.6)	5 (0.7)	81 (1.5)
Other/not specified	2068 (44.1)	300 (43.7)	2368 (44.1)
Lumbar spine DEXA categories *, *n* (%)			
Normal (≥−1.0)	396 (51.9)	102 (50.0)	498 (51.5)
Osteopenia (−1.0 to −2.5)	273 (35.8)	85 (41.7)	358 (37.0)
Osteoporosis (≤−2.5)	94 (12.3)	17 (8.3)	111 (11.5)
Femoral neck DEXA categories *, *n* (%)			
Normal (≥−1.0)	321 (51.2)	95 (47.0)	416 (50.2)
Osteopenia (−1.0 to −2.5)	261 (41.6)	92 (45.5)	353 (42.6)
Osteoporosis (≤−2.5)	45 (7.2)	15 (7.4)	60 (7.2)

Abbreviation: DEXA, dual-energy X-ray absorptiometry. * Data were from all scans throughout the enrolled period. Individual patients may contribute data from multiple scans. DEXA categories were assessed using T-scores for all patients aged ≥21 years.

**Table 4:** Summary of AEs by treatment status.

AEs, *n* (%); No. of Events	Treated (*n* = 1643)	Untreated (*n* = 441)	Total (*N* = 2084)
AEs	552 (33.6); 1459	29 (6.6); 40	581 (27.9); 1499
Velaglucerase alfa–related AE *	29 (3.3); 41	0	29 (3.3); 41
SAEs	340 (20.7); 783	24 (5.4); 32	364 (17.5); 815
Velaglucerase alfa–related SAE	2 (0.2); 2	0	2 (0.1); 2 ^†^
Fatal AEs ^‡^	79 (4.8); 96	8 (1.8); 8	87 (4.2); 104
Most frequent AEs (>1%)			
COVID-19	40 (2.4); 40	2 (0.5); 2	42 (2.0); 42
Arthralgia	29 (1.8); 36	0	29 (1.4); 36
Headache	24 (1.5); 26	0	24 (1.2); 26
Back pain	22 (1.3); 24	1 (0.2); 1	23 (1.1); 25
Abdominal pain	22 (1.3); 23	0	22 (1.1); 23

Abbreviations: AE, adverse event; GOS, Gaucher Outcome Survey; *n*/*N*, number of patients; SAE, serious adverse event. AEs defined as any event occurring during the follow-up period. * Velaglucerase alfa–related events as a proportion of patients treated with velaglucerase alfa (*n* = 886). ^†^ Both events were osteonecrosis. ^‡^ Fatal AEs were not captured in the GOS for 10 deaths.

## Data Availability

The datasets, including the redacted study protocol, redacted statistical analysis plan, and individual participants’ data supporting the results reported in this article, will be made available within 3 months from initial request to researchers who provide a methodologically sound proposal. The data will be provided after its de-identification, in compliance with applicable privacy laws, data protection, and requirements for consent and anonymization.

## Supplementary Materials

The following supporting information can be downloaded at: https://www.mdpi.com/article/10.3390/jcm13123588/s1, Table S1: Mutations identified as ‘other’; Table S2: Classification of medical history events. Events included in the GED-C PSS for diagnosis of GD [43]. Blue: Major signs and covariables. Orange: Minor signs and covariables.
