# Supplementary material for: Twelve Years of the Gaucher Outcomes Survey (GOS): Insights, Achievements, and Lessons Learned from a Global Patient Registry
Source: J Clin Med. 2024 Jun 19;13(12):3588. doi: 10.3390/jcm13123588 (PMC11204885; doi:10.3390/jcm13123588)
Supplement: Supplementary file 1 [file jcm-13-03588-s001.zip › jcm-2997111-supplementary.pdf]

Supplementary Materials

Table S1. Mutations identified as 'other'

Gaucher disease type	Treatment status	Classification coded	Specific mutation identified
1	Treated	Other/Other	R463C/R463C
1	Treated	Other/Other	84GG, and S356F
1	Treated	Other/Other	p.R496H/c.84dupG
	Treated	Other/Other	c.1171G>C/c.1192C>T
1	Treated	Other/Other	c.259C>T/RecNci1
1	Treated	Other/Other	c.259C>T/c.259C>T
1	Treated	Other/Other	R496H/8466
1	Untreated	Other/Other	pW330X/R496H
1	Treated	Other/Other	E349K/S366N
1	Treated	Other/Other	R496H/84GG
3	Treated	Other/Other	c.1342G>C/c.1504C>T
1	Treated	Other/Other	RecNcil/A338T
1	Treated	Other/Other	84GG other mutation unknown
1	Treated	Other/Other	84GG and unknown
1	Treated	Other/Other	N370S/N370S
1	Treated	Other/Other	c.245C>T/c.245C>T
1	Treated	Other/Other	p.R496H/84dupG
1	Treated	Other/Other	p.R463C/p.N462K
1	Treated	Other/Other	F397S/c.334_338delCAGAA
1	Treated	Other/Other	Not available
1	Treated	Other/Other	RecNci1/p.R48W
1	Treated	Other/Other	Not available
1	Treated	Other/Other	V391L, L483Q
1	Treated	Other/Other	E394K/S366N

2	Untreated	Other/Other	p.R159W/ p.R502C c.475C>T / c.1504C>T
1	Treated	Other/Other	R202X and D448G
	Untreated	Other/Other	c.84dupG/c.259C>T
1	Treated	Other/Other	84GG/R48W
1	Treated	Other/Other	R48W/R48W
1	Treated	Other/Other	G377S/G377S
3	Treated	Other/Other	R463C/960-964 del GGATG
1	Untreated	Other/Other	R87W/R87W
1	Treated	Other/Other	R87W/R87W
1	Treated	Other/Other	R463C/IVS2+1G>A
1	Treated	Other/Other	R496H/R359X
1	Treated	Other/Other	R463C [R502C] / L105R
1	Treated	Other/Other	R463C/c.595_596 het_del CT + S271G
1	Treated	Other/Other	R48W/R48W
1	Treated	Other/Other	N396T/N396T
1	Untreated	Other/Other	N396T/N396T
1	Treated	Other/Other	RecNcil/R48W
1	Treated	Other/Other	R48W/RecNcil
1	Treated	Other/Other	RecNci/R48W
1	Treated	Other/Other	L483P/L444P
1	Treated	Other/Other	G377S/G377S
1	Treated	Other/Other	R496H/IVS2+1
1	Treated	Other/Other	R496H/R496H
1	Treated	Other/Other	R946H/84GG
1	Treated	Other/Other	W184R/V375L
1	Treated	Other/Other	84GG, R486H
1	Untreated	Other/Other	R87W/R87W
1	Untreated	Other/Other	R87W/R87W
2	Untreated	Other/Other	D409H/VUS
1	Treated	Other/Other	R48W/RecNcil1

1	Treated	Other/Other	D409H/H255Q:A377T
2	Treated	Other/Other	V394L/84GG
1	Treated	Other/Other	D409H;H255Q
	Treated	Other/Other	D409H;H255Q/D409H;H255Q
3	Treated	Other/Other	F213I/D409H;H255Q
1	Treated	Other/Other	D409H;H255Q
1	Treated	Other/Other	c.(259C>T); (1265_1319del55)
1	Treated	Other/Other	G46E/R257Q
	Treated	Other/Other	L483P/L483P
	Untreated	Other/Other	IVS2+1/R496H
1	Treated	Other/Other	84GG/R496H
1	Treated	Other/Other	c.(259C>T);(1265_1319del55)
3	Treated	Other/Other	R463C/G377S
1	Treated	Other/Other	Not available
2	Untreated	other/other	R159W/E272D
2	Treated	Other/Other	K237E
3	Treated	other/other	IVS2+1G>A/N188S
3	Treated	Other/Other	K118N/D448H
	Treated	other/other	N448H/T69Dfs*12
1	Untreated	Other/Other	R496H/R496H
3	Treated	Other/Other	L483P Heterozygous
3	Treated	Other/Other	D409H/D409H
3	Treated	Other/Other	D409H/D409H
3	Treated	Other/Other	D409H/D409H
	Treated	Other/Other	c.203del/L483P
1	Untreated	Other/Other	R496H/84GG
1	Treated	Other/Other	R48W/R48W
	Untreated	Other/Other	N409S

Table S2. Classification of medical history events. Events included in the GED-C PSS for diagnosis of GD [1]. Blue: Major signs and covariables. Orange: Minor signs and covariables.

Abdominal & GI/GU

Abdominal pain
Abnormal LFTs
Abscesses (all sorts) pancreatitis
Accessory spleen enlargement
Ano-rectal fissurae ani
Barrett's esophagitis
Benign prostatic hypertrophy (BPH)
Biliary colic
Cholecystitis
Cholelithiasis /vesicular lithiasis /gallstones
Cirrhosis
Colitis
Constipation (chronic)
Crohn's disease
Cysts (all sorts)
Diarrhea (chronic)
Diverticulosis
Dysphagia
Early satiety
Eosinophilic Esophagitis (EoE)
Esophageal varices
Fibrosis
Gastroesophageal Reflux Disease (GERD)
Gilbert's syndrome
Gout
Hematuria
Hemorrhoids (internal/external)
Hepatomegaly
Hepatopulmonary syndrome
Hernias (all sorts)
Hyperbilirubinemia
Hypospadias
Incontinence
Indigestion
Infarcts/lesions
Inflammatory bowel disease (IBD)
Irritable bowel syndrome (IBS)
Jaundice
Melena
Meniere's disease
Nausea
Non-alcoholic fatty liver disease (NAFLD)
Non-alcoholic steatohepatitis (NASH)
Pollakiuria (day-time wetting)
Polyps (all locations)
Polyuria
Portal hypertension
Portal vein thrombosis
Prostatitis
Rectal bleeding
Splenomegaly
Ulcerative colitis
Ulcers (peptic/gastric)

Urinary Tract Infection (UTI)
Vomiting

Allergies

Additives
Atopic dermatitis
Drugs (sulfa/penicillin)
Egg allergy
Fruits / vegetables
Gluten intolerance
Hay fever
IgE elevated
Lactose intolerance
Seasonal allergies
Soy allergy
Textiles

Cancers and tumors

Basal cell carcinoma (BCC)
Bladder
Breast
Colon
Glioblastoma
Hepatocellular carcinoma (HCC)
Lymphoma
Melanoma
Multiple myeloma (MM)
Myelodysplastic syndrome (MDS)
Ovarian
Pancreas
Prostate
Rectal
Renal
Sarcoma
Suprasellar lesions (assuming a neoplasm)
Squamous cell
Testicular
Uterine

Cardiovascular

Angina
Aortic valve stenosis
Bradycardia
Cavernoma
Chest pains
Coronary artery bypass grafting (CABG)
Cerebrovascular accident (CVA)
Edema
Hypertension (systemic)
Ischemic heart disease
Left ventricular hypertrophy (LVH)
Mitral valve prolapse (MVP: Barlow’s)
Myocardial infarction (MI)
Orthostatic hypertension
Palpitations
Patent Ductus Arteriosus (PDA)
Patent Foramen Ovale (PFO)
Tachycardia
Systolic murmurs
Transient Ischemic Attack (TIA)
Valve (mitral/aortic) calcifications
Varicose veins

Deficiencies

Calcium, magnesium, phosphor, electrolytes
Folic acid
Iron
Vitamin B12
Vitamin D

Dermatology

Acne
Alopecia
Café-au-lait spots
Condiloma
Contact dermatitis
Cysts (all sorts)
Eczema
Hemangioma
Hyperpigmentation
Lipoma
Nevii (pigmented)
Palmar/plantar erythema
Pityriasis rosea
Pruritis
Psoriasis
Rashes
Rosacea
Seborrhea
Stasis dermatitis
Ulcers
Urticaria /itching
Verruca/warts
Versicolor tinae/pityriasis
Vitiligo

EENT

Adenoid hypertrophy
Amaurosis fugax
Aphthous (canker) sores
Aphthous infections
Blepharitis
Blindness
Cataract
Conjunctivitis
Corneal opacities
Dental caries/broken teeth
Dry eyes
Eardrum perforation
Eye infections
Hearing Loss
Hyperopia (far-sightedness)
Macroglossia
Macular degeneration
Meniere’s disease
Meibomitis (dry eyes)
Myopia
Nystagmus

Oculomotor apraxia (OMA)
Otitis media /glue ear
Periodontal disease
Pinguculae
Pre-retinal opacities (white dots)
Pterygium
Ptosis
Retinal detachment
Sinusitis
Strabismus
Supranuclear horizontal gaze palsy (SHGP)
Tempero-mandibular Junction (TMJ) pain
Tinnitus
Tonsilitis
Trichiasis (eyelashes)
Uveitis
Vocal cord damage

Endocrine

Adrenal insufficiency
Diabetes insipidus
Diabetes mellitus (type 1/type 2)
Goiter
Graves disease
Growth hormone (GH) deficiency
Hashimoto's thyroiditis
Height/growth retardation
Hot flushes/flashes
Hyperparathyroidism
Hyperthyroid
Hypogonadism
Hypothyroid
Menopause
Metabolic syndrome
Night sweats
Polydypsia
Pubertal delay
Thyroid Stimulating Hormone (TSH) elevated

General

Abnormal lab values: ACE, TRAP, alkaline phosphatase, acid phosphatase
Addiction (narcotic / recreational)
Asthenia
Cachexia
Failure to thrive (FTT)
Fatigue
Fungus
Hair loss
Hypercholesterolemia
Hypertriglyceridemia
Iron overload
Malaise
Pain (no other descriptive)
Sexually transmitted disease (STD)
Smoking cigarettes/pipe
Weakness
Weight gain / obesity
Weight loss / anorexia
Worms

Hematology

Anemia
Antiphospholipid syndrome / antiphospholipid antibody syndrome (APS / APLA)
Clotting factor deficiency
Coagulation factor deficiencies (including all)
Deep vein thrombosis (DVT)
Ecchymoses
Easy bruising
Eosinophilia
Epistaxis
Gingival bleeding
Hemolytic anemia
Hemorrhage (not post-partum)
Hematomas
Hyperferritinemia
Immune thrombocytopenic purpura (ITP)
Leukopenia
Macrocytosis
Methylene tetrahydrofolate reductase (MTHFR) deficiency
Microcytosis
Neutropenia
Pancytopenia
Petechiae
Platelet function abnormality
Polycythemia vera
Protein S
Prothrombin time (PT) / activate partial thromboplastin time (aPTT) prolonged
Tendency to bleed
Thrombocytopenia
Thrombocytosis
Transfusion dependency
von Willebrand factor/von Willebrand disease

Immune

Anti-transglutaminase antibodies (ATA)
Autoimmune hemolytic anemia (Coombs positive)
Autoimmune/inflammatory syndrome induced by adjuvants (ASIS; Schonfeld's)
Biclonal gammopathy
Lupus erythematosus (systemic / dermatologic)
Monoclonal gammopathy
Monoclonal gammopathy of undetermined significance (MGUS)
Paraproteinemia
Polyclonal gammopathy
Reactive arthritis
Rheumatoid factor increased
Scleroderma
T-cell abnormality

Infections and inflammation

Ankylosing spondylitis
Appendicitis
Arthritis (no descriptives)
Bronchitis
Candidiasis

Cellulitis
Costochondritis / tietze syndrome
Cytomegalovirus (CMV)
Epstein-Barr Virus (EBV; mononucleosis)
Fibromyalgia
Flatfoot
Helicobacter pylori infection
Hepatitis A/B/C
Herpes / Herpes zoster
Hypotonus
Interstitial cystitis (bladder)
Leishmaniasis
Leptospirosis
Lyme disease
Malaria
Measles
Meningitis
Mumps
Norovirus
Osgood-Schlatter (patellar) disease
Osteomyelitis
Pancreatitis
Q fever
Rheumatic fever
Rheumatoid arthritis
Rubella
Sacroiliitis
Sepsis
Stomatitis
Torticollis
Tuberculosis

Vaginitis
Varicella (chickenpox) /zoster
Vestibulitis

Lymph

Single node enlargement
Lymphadenopathy (any location)

Miscellaneous inherited conditions

Alpha-thalassemia
Branchio-Oto-Renal (BOR) syndrome
Color blindness
Cystic fibrosis (CF)
Down syndrome
Ehlers-Danlos
Galactosemia
Haglund’s deformity
Hemochromatosis
Lhermitte’s sign / multiple sclerosis
Neurofibromatosis
Paget’s disease
Wolff-Parkinson-White
Xeroderma pigmentosa

Muscles and joints

Arthropathy
Arthrosis
Baker’s cyst
Bunions
Bursitis
Disc (spinal) pathology

Enthesopathy (ligament)
Flatfeet / Plattfuss
Frozen shoulder
Hemarthrosis
Joint pain
Ligament tears
Meniscus (knee) tears
Muscle atrophy
Muscle cramps
Muscle pain
Opisthotonus
Osteophytes
Polyarthritis
Psoriatic arthritis
Rheumatoid arthritis
Rotator cuff (shoulder) injury
Sarcopenia
Sciatica (pain)
Spinal stenosis
Tennis elbow
Trigger finger
Whiplash injuries

Neurologic/psychiatric

Anxiety
Apnea
Asperger’s
Attention deficit disorder (ADD)
Attention deficit hyperactivity disorder (ADHD)
Autism
Bell’s palsy
Bipolar disease
Breath-holding
Burning sensation in an appendage / digits
Carpal-tunnel syndrome
Charcot-Marie-Tooth syndrome
Chronic fatigue syndrome
Cognitive decline/memory impairment
Delusions
Depression
Dyspraxia / Developmental Coordination disease
Epilepsy
Headaches
Hereditary Neuropathy with Liability to Pressure Palsy (HNPP)
Insomnia /sleep disorder
Meningioma
Migraines
Morton’s neuroma
Myoclonus
Nightmares
Numbness
Panic attacks
Paresis
Peripheral neuropathy
Pervasive development disorder (PDD)
Presyncope
Psychosis
Radiculopathy
Restless legs syndrome
Reynaud’s phenomenon
Seizures
Sleep apnea

Spasticity
Suicidal ideation / attempts
Syncope
Tic disease (twitching)
Tingling in an appendage/digits
Tremor
Vertigo

Conditions related to Parkinson's disease and Lewy body disease

Ataxia /gait impairment
Cognitive decline
Color discrimination impairment
Dementia
Hallucinations
Sleep disturbances
Smell impairment
Speech impairment
Tremor

Pulmonary

Abnormal pulmonary function test (PFT)
Asthma
Chronic cough
Chronic obstructive pulmonary disease (COPD)
Cyanosis
Digital clubbing
Dyspnea / shortness of breath
Exertional dyspnea
Infiltrative lung disease
Interstitial lung disease /markings
Pleural effusion
Pneumothorax
Pulmonary hypertension (PHT)
Upper respiratory infections (URI) / recurrent

Renal

Acute kidney injury (AKI)
Acute tubular necrosis (ATN) disease
Atrophic kidney
Chronic kidney disease
Dysplastic kidney(s)
Dysuria
Glomerulonephritis
Hypercalcemia
Hypercalciuria
Hyperuricemia
Hyperuricosuria
Hypoalbuminemia
Nephrolithiasis
Polycystic kidney disease
Proteinuria
Pyelonephritis
Renal colic

Reproductive (female and male)

Breast fibroma
Cysts (all sorts in sex organs)
Endometriosis
Epididymis varicocele
Funiculocele

Gestational diabetes
Gestational thrombocytopenia
Hydrocele
Infertility (female/male)
Leiomyoma
Mastopathy
Menometrorrhagia/ Menorrhagia
Menorrhagia
Oligospermia
Pelvic inflammatory disorder (PID)
Polycystic ovaries
Polyps (all sorts)
Post-partum hemorrhage
Recurrent abortions
Undescended testicles
Uterine fibroma

Skeletal

Avascular necrosis / osteonecrosis/ joint replacement
Bone crisis
Erlenmeyer flask deformity of distal femur
Fractures (all locations)
Herring bone /Chevron sign of humerus
Kyphosis
Lordosis
Lytic lesions/infarcts
Marrow infiltration
Osteoarthritis / degenerative changes / arthrosis
Osteoporosis/osteopenia
Pectus excavatum
Perthes (Legg-Calve-Perthes) disease

Sclerosis (including spine)
Scoliosis
Spina bifida
Traumatic injury (e.g., whiplash)
Valgus/varus deformity
Vertebral collapse

Reference

1. Mehta, A.; Kuter, D.J.; Salek, S.S.; Belmatoug, N.; Bembi, B.; Bright, J.; Vom Dahl, S.; Deodato, F.; Di Rocco, M.; Göker-Alpan, O.; et al. Presenting signs and patient co-variables in Gaucher disease: outcome of the Gaucher Earlier Diagnosis Consensus (GED-C) Delphi initiative. *Intern Med J* **2019**, *49*, 578-591.
